# Optimization of Liquid Culture Condition of a Novel Fungus *Hygrophoropsis* sp. and Antioxidant Activity of Extracts

**DOI:** 10.1155/2020/7403257

**Published:** 2020-08-01

**Authors:** Liang Huang, Chunxia Li, Ning Sun, Yu Wang, Hongpeng Yang, Yiting Li, Litong Ban

**Affiliations:** College of Agronomy and Resources Environment, Tianjin Agricultural University, Tianjin 300384, China

## Abstract

To evaluate pharmacological activities of a novel fungus *Hygrophoropsis* sp., the influence of aeration rate on the production of mycelial biomass, exopolysaccharides (EPS), and intrapolysaccharides (IPS) in the fungus *Hygrophoropsis* sp. was investigated. And the water extracts of cultured *Hygrophoropsis* sp. mycelia and the fermentation broth were analyzed for their antioxidation activity by using four different assay methods such as hydroxyl radical scavenging, superoxide radical scavenging, hydrogen peroxide scavenging, and reducing power. The *Hygrophoropsis* sp. was cultivated under various aeration rates in a 7 l bioreactor. The highest mycelial biomass (3.98 mg/mL) and IPS production (19.63 mg/g) were obtained at aeration rate 4.5 v.v.m. The results showed that *Hygrophoropsis* sp., in general, possesses a strong antioxidation activity in all assays tested. The deproteinized extracts had stronger antioxidation activity as compared to the un-deproteinized extracts by using superoxide radical scavenging, hydrogen peroxide scavenging, and reducing power. Besides, the un-deproteinized extracts had stronger antioxidation activity as compared to the deproteinized extracts by using hydroxyl radical scavenging. Thus, the polysaccharide extractions from the *Hygrophoropsis* sp. studied have antioxidant activities *in vitro*, which may be a good source of natural antioxidants or further investigation as potential natural antioxidants.

## 1. Introduction

Some synthetic antioxidants, such as butylated hydroxyanisole and butylated hydroxytoluene, are used for food additives but not in medical health because of their toxicity [1]. Mushrooms are increasingly attractive as natural, nontoxic antioxidants and as potential sources of physiologically important components [2–4]. Polysaccharides are an important component of various types of fungi, a major factor in their bioactive properties, such as the suppression of damage caused by cytostatics and radiation to the immune system cells [5–8].

**Figure 1:**
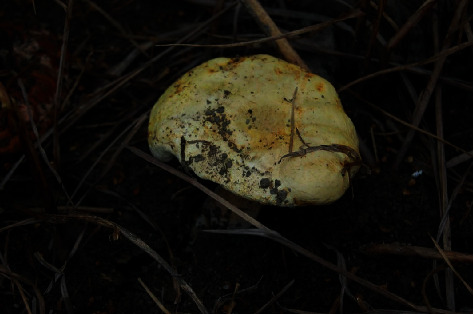
Wild fungus “Weimo” among reeds.

A novel fungus called “Weimo” in Ninghe District Tianjin Province was found among reeds. The fruiting body is yellow, like butter (Figure 1). This wild mushroom is rare but tasty, which was sold for 4000 RMB per kilogram. Mycelia isolated from fruiting body tissues were cultured and preserved on slant medium in our laboratory. It was identified as *Hygrophoropsis* sp. belonging to Hygrophoropsidaceae family by ITS sequence.

This edible fungus is hard to cultivate for fruiting bodies but only the right environment and special substance are available. However, fungal mycelia can easily be acquired in sufficient quantities by fermentation in order to produce biologically active supplements, and the submerged cultivation method is an acceptable method [4].

In comparison with the commercial mushroom products, mycelia or fermentation broth has many advantages such as shorter culture period, consistent product quality, and independent of seasonality [9–11]. *Hygrophoropsis* sp. mycelia were obtained by submerged cultivation in a batch system from shaking flask to fermenter. The *in vitro* antioxidative activities of the extracts were tested by using different assay methods. The biological activities of the crude polysaccharides suggested it is applicable for resources development.

## 2. Materials and Methods

The fruiting body of “Weimo” was picked in Ninghe District, Tianjin province. Cultured mycelia were obtained from tissues and preserved at Technology Research and Development Center for Edible Fungus, Tianjin Agricultural University, Tianjin, China.

### 2.1. Preparation of Liquid Medium

The mycelia was cultured in the liquid medium with 95 mL working volume in 250 mL triangular flask at 28°C, which consisted of 15 mg/mL glucose, 10 mg/mL sucrose, and 4 mg/mL yeast powder.

### 2.2. Liquid Fermentation

The 4 L liquid fermentation medium was added to a 7 L fermenter which has been already sterilized. Then, the medium was sterilized at 121°C for 20 min again. After the strain seed culture was added, the aeration rate was set at 3, 4.5, and 6 L/min, respectively. The mycelia were collected at different intervals. Samples were centrifuged at 5000 rpm for 10 minutes; the supernatant and precipitate were preserved, respectively, for polysaccharides determination.

### 2.3. Isolation of IPS and EPS

#### 2.3.1. Extraction of IPS

The extraction method of polysaccharides was performed according to the procedure described by Zhang et al. [12], with some modifications. The IPS solution was extracted from dried mycelia filtered by hot water with 1 : 10 ratio, which was precipitated by adding 4 times of ethanol (90%) subsequently. The precipitate was suspended in water and centrifuged to remove insoluble material.

#### 2.3.2. Extraction of EPS

The supernatant was concentrated to 1/2 of the original volume by rotary evaporation under reduced pressure at 55°C. The concentrated EPS solution was treated with 4 times of ethanol and then centrifuged at 5000 rpm for 10 min to get the precipitate. The precipitate was suspended in water and centrifuged to remove insoluble material [13].

### 2.4. Purification of Polysaccharides

Deproteinization procedure was carried out according to reference [14] with some modifications as follows: The crude polysaccharides solution was added 4 times the volume of savage reagent (chloroform : normal butanol = 5 : 1), shaken violently for 30 min, and then rested for 10 min. The intermediate protein layer was removed from the mixture which was centrifuged for 10 min. Moreover, the supernatant solution was preserved. The procedure was repeated 3–5 times to ensure that all proteins are removed.

Un-deproteinized polysaccharides were purified without deproteinization procedure but further procedure [15], while deproteinized polysaccharides were subsequently purified as follows: the supernatant solution was added 4 times the volume of ethanol and kept for 12 h at 4°C; the precipitation was the purified polysaccharides after centrifuging at 5000 rpm for 10 min. Furthermore, polysaccharides were washed with ethanol for 2-3 times, and then lyophilized; the polysaccharides sample was obtained finally.

### 2.5. Determination of the Glucose Standard Curve

The yield of polysaccharides was determined by phenol-sulfuric acid method as in reference [16] with some modifications. The standard glucose solution was prepared (the glucose was dried to the constant weight) with a concentration of 0.04 mg mL^−1^. The glucose solution was taken as 0 (reference), 0.2, 0.3, 0.4, 0.5, 0.6, 0.7, and 0.8 mL, respectively, which were complemented to 1.0 mL with distilled water. Then, they were shaken after adding phenol (5%) 0.5 mL and sulfuric acid 2.5 mL. The absorbance was measured at 490 nm after 30 minutes at room temperature. The standard curve was drawn with the polysaccharides concentration as the horizontal coordinate and the absorbance value as the ordinate.

The polysaccharides sample (1 mL) substituted for the glucose solution was determined according to the above method.

### 2.6. Extraction and Optimization of IPS

The IPS was extracted with water extracting-alcohol precipitating. The IPS (1 g) was extracted by rotating evaporator, and the supernatant was used for the determination. The orthogonal experiment of L_9_ (3^3^) was designed with the solid-liquid ratio, extraction time, and extraction temperature as the factors. The levels of the factors are shown in Table 1.

#### 2.6.1. Calculation of Extraction Rate of Polysaccharides

The extraction rate of polysaccharides was calculated as follows. The extraction rate of polysaccharides (%) = *X* × *V*/*M* × 100%, where *X* is sample polysaccharides concentration (mg/mL), *V* is sample volume (mL), and *M* is mass of mycelia (*g*).

#### 2.6.2. Alcohol Precipitation of Polysaccharides

Aqueous polysaccharides of 5 mL were added with ethanol to the final concentration of ethanol solution 60%, 65%, 70%, 75%, 80%, 85%, and 90% (v/v) and sat quietly for 24 h. The optimum ethanol concentration was obtained by the content of polysaccharides.

### 2.7. Determination of Antioxidant Capacity

#### 2.7.1. Ability of Scavenging Hydroxyl Radicals

The ability of scavenging hydroxyl radicals of sample polysaccharides was determined according to the principle of the Fenton reaction system as in reference [17] with some modifications. The reaction system consisted of phosphate buffer solution (pH 7.4) 0.5 mL, Saffron solution (0.5%) 0.1 mL, EDTA Na_2-_Fe^2+^ solution (0.1 mol/L) 0.5 mL, sample polysaccharides 3.5 mL, and H_2_O_2_ (6%) 0.4 mL. The absorbance was measured at 520 nm after a water bath at 40°C for 30 min. The blank was done as follows: sample polysaccharides solution was replaced by distilled water of equal volume, and the control group, polysaccharides solution, and EDTA Na_2_-Fe^2+^ solution were replaced in the same way. The scavenging rate of hydroxyl radicals was calculated as follows: Hydroxyl radicals scavenging rate (%) = (1 − (Δ*A*_520_ of sample − Δ*A*_520_ of blank)/(Δ*A*_520_ of control − Δ*A*_520_ of blank)) × 100%.

#### 2.7.2. Ability of Scavenging Superoxide Anion Radicals

The ability of scavenging superoxide anion free radicals (O^2−^) was determined according to the principle of pyrogallol autooxidation [18]. Each sample of polysaccharide solution with different concentrations (0.2 mL) was mixed with Tris-HCl (pH 8.2) buffer solution (5.6 mL). Then, pyrogallol solution (preheat at 25°C, 30 mmol/L, 0.2 mL) was added after the water bath at 25°C for 10 min. The reaction of the mixture was accurate 30 min after shaking vigorously. Finally ascorbic acid (*V*_*C*_) solution (5%, 0.1 mL) was added to terminate the reaction. The absorbance was measured at 420 nm after resting for 10 min. In control group, 0.2 mL distilled water was used to replace the sample, and in blank group, 5.6 mL Tris-HC1 solution was used to replace the pyrogallol solution. The scavenging rate of superoxide anion radicals was calculated as follows: Superoxide anion radicals scavenging rate (%) = ((Δ*A*_420_ of control − Δ*A*_420_ of sample)/Δ*A*_420_ of control) × 100%.

#### 2.7.3. Reducing Power

The Oyaizu [19] method was used to determine the reduction force of the sample polysaccharides with minor modifications. The different concentrations of polysaccharides solution (1 mL) were mixed with phosphate buffer solution (pH 6.6, 2.5 mL) and 1% potassium ferricyanide solution (2.5 mL). The mixture was shaken and centrifuged for 10 min at 800 rpm. The supernatant (2.5 mL) was mixed with distilled water and 0.1% ferric chloride solution (2.5 mL, respectively). The absorbance was measured at 700 nm against a blank after shaking and resting for 10 min.

#### 2.7.4. Ability of Scavenging 1, 1-Diphenyl-2-picrylhydrazyl (DPPH) Radicals

DPPH free radical scavenging activity was measured according to the method of Kao and Chen [20], with some modifications. The polysaccharides solution with different concentrations (3 mL) was mixed with DPPH solution (0.004% in ethanol, 1.0 mL). The mixture was shaken vigorously and rested for 30 min in the dark, and the absorbance was then measured at 517 nm against the blank. *V*_C_ was used for positive control. The scavenging rate of DPPH radicals was calculated as follows: DPPH radicals scavenging rate (%) = ((Δ*A*_517_ of blank – Δ*A*_517_ of sample)/Δ*A*_517_ of blank) × 100%.

### 2.8. Statistical Analysis

Statistical analysis involved the use of the statistical analysis system SPSS v.17. 0 program. Analysis of variance was performed by ANOVA procedures. Each item of data represents mean values and standard deviation (SD) of three replicates. Significant differences between the two means were determined by Tukey's test. *P* values <0.05 were regarded as significant.

## 3. Results

### 3.1. Glucose Standard Curve

**Figure 2:**
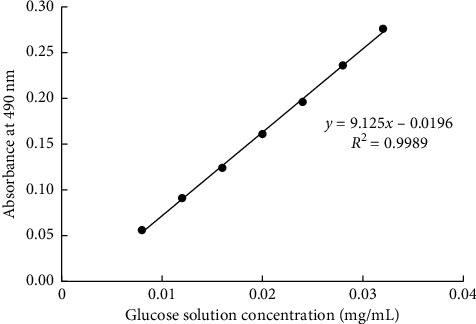
Glucose standard curve.

The glucose standard curve is shown in Figure 2. *R*-square is 0.9989, which revealed good linear relation between absorbance and glucose solution concentration.

### 3.2. Effect of Aeration Rate on the Intracellular Polysaccharide (IPS) and Exopolysaccharide (EPS) Yield

**Figure 3:**
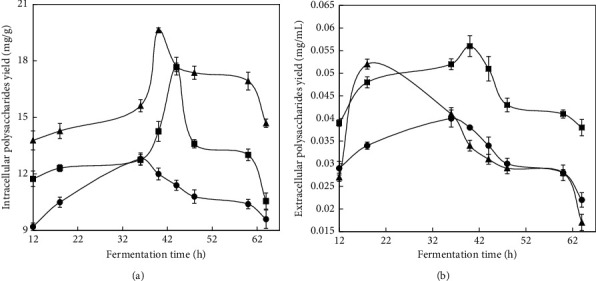
Time profiles of (a) intracellular polysaccharide (IPS) yield and (b) exopolysaccharide (EPS) yield in *Hygrophoropsis* sp. using a 7-l stirred tank reactor at different aeration rates; (●) 3 L/min; (▲) 4.5 L/min; (■) 6 L/min. Each value is a mean of three biological replicates.

The effect of aeration rate on IPS and EPS production is shown in Figure 3. The yield of IPS production was higher than that of EPS. The yield of IPS was the highest (19 mg/g) at 40 h with the aeration rate 4.5 L/min, while EPS production reached the highest rate (0.056 mg/mL) at 40 h with the aeration rate 6 L/min.

### 3.3. Optimization of Extraction Conditions for IPS Production

The range *R* is applied to estimate the magnitude of influence of each parameter [21]. According to the results of orthogonal analysis in Table 2, the magnitude of influence for the extraction of IPS from *Weimo* mycelia can be sorted by *A* (Solid-liquid ratio) > *B* (Extraction time) > *C* (Extraction temperature). There were significant differences among the three factors on the extraction of IPS from *Weimo* mycelia (Table 3) and in contrast to *R* values (Table 2), it can be found that the parameter solid-liquid ratio is the most significant parameter for the extraction of IPS from *Weimo* mycelia, and extraction temperature is the least important parameter.

The optimal value of specific parameter can be determined based on the results of K_*i*_ in Table 2. In the present research, the optimum values for each parameter are as follows: *A* = 1 : 30, *B* = 2, *C* = 85. Under the optimum extraction method (A1B2C1) of IPS based on the orthogonal optimization method, the extraction rate of IPS was 11.36%. The extraction rate and yield of IPS were higher than those of group *A*1*B*1*C*1, which validates the reliability of design method and the optimization strategy for IPS extraction that is proposed in the present research.

### 3.4. Effect of Ethanol Concentration on the Precipitation of Polysaccharides

**Figure 4:**
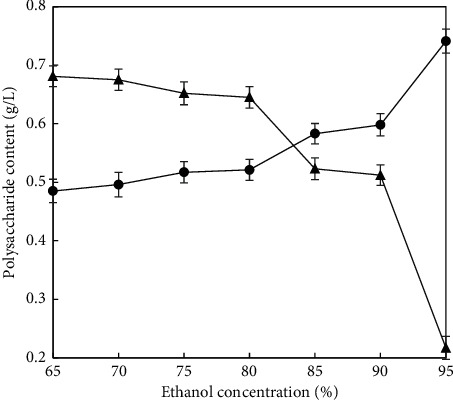
Effect of ethanol concentration on polysaccharides precipitation; (●) polysaccharides in precipitation; (▲) polysaccharides in supernatant. Each value is a mean of three biological replicates.

The yield of polysaccharides in supernatant gradually decreased, and the yield of polysaccharides in precipitate increased gradually with the increase of ethanol concentration (Figure 4). When the concentration of ethanol was in the range of 80–85%, the yield of polysaccharides in supernatant decreased, instead, and the yield of polysaccharides in precipitation increased obviously. The yield of polysaccharides in supernatant and precipitate tended to be stable at 85–90%. However, the yield of polysaccharides in precipitation continued to increase, when ethanol concentration reached 95%. On the premise of ensuring maximum precipitation of polysaccharides and saving reagents, 80–85% ethanol was selected for experiment. Within this range, the yield of polysaccharides in precipitation was 0.52–0.58 mg/mL, as the yield of polysaccharides in supernatant was 0.65–0.52 mg/mL.

### 3.5. Determination of Antioxidant Activity

#### 3.5.1. Scavenging Ability of Hydroxyl Radicals

**Figure 5:**
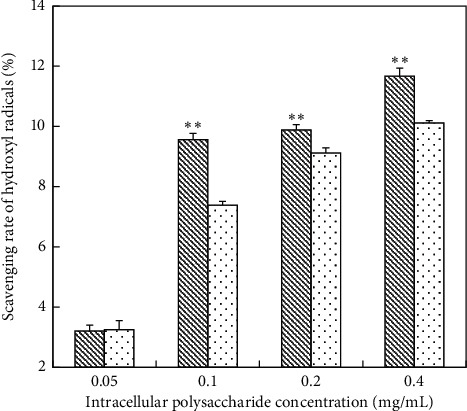
Scavenging hydroxyl radical activity of IPS; (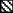) un-deproteinized IPS; (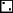) deproteinized IPS. The significance in difference between un-deproteinized IPS and deproteinized IPS was calculated by the paired-samples *T* test (^*∗∗*^*P* < 0.01).

*(1) Scavenging Ability of Hydroxyl Radicals on IPS*. The scavenging ability of hydroxyl radicals on un-deproteinized IPS was stronger than the deproteinized IPS (Figure 5). When the content of un-deproteinized IPS was at 0.05–0.1 mg/mL, the scavenging rate of hydroxyl radicals increased significantly from 3.21% to 9.56%. The scavenging rate of hydroxyl radicals was significantly different when the content of un-deproteinized IPS and deproteinized IPS was at 0.1–0.4 mg/mL. With regard to un-deproteinized IPS, the scavenging rate was from 9.56% to 11.67% within the above concentration range, while the scavenging rate of deproteinized IPS was from 7.39% to 10.12%. The scavenging rate of hydroxyl radicals increased little in the range of 0.1–0.4 mg/mL un-deproteinized IPS, and the maximum scavenging rate of hydroxyl radicals reached 11.67% at the content of un-deproteinized IPS 0.4 mg/mL. Compared with VC solution, both of the un-deproteinized IPS and deproteinized IPS were lower than VC solution in scavenging hydroxyl radicals. When VC solution was at 0.4 mg/mL, the scavenging rate of hydroxyl radicals reached 47%, which was far more than the scavenging rate of two kinds of polysaccharides solution at 0.4 mg/mL (Table 4). With regard to the deproteinized IPS, the scavenging rate of hydroxyl radicals increased gradually during the enrichment of crude polysaccharides. The scavenging rate of hydroxyl radicals was 10.12% when the content of deproteinized IPS was 0.4 mg/mL.

**Figure 6:**
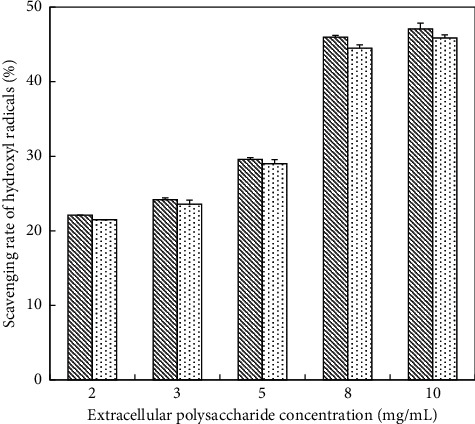
Scavenging hydroxyl radical activity of EPS; (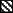) un-deproteinized EPS; (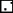) deproteinized EPS. The significance in difference between un-deproteinized EPS and deproteinized EPS was calculated by the paired-samples *T* test (^*∗∗*^*P* < 0.01).

*(2) Scavenging Ability of Hydroxyl Radicals on EPS*. With regard to EPS, there is no significant difference between the un-deproteinized EPS and deproteinized EPS. However, the scavenging rate of un-deproteinized EPS was slightly higher than deproteinized EPS (Figure 6). The scavenging ability of hydroxyl radicals strengthened with the increasing concentrations of EPS (Figure 6). It is obvious that the scavenging rate tended to be stable when the concentrations of EPS were from 8 to 10 mg/mL. In addition, the maximum scavenging rate (the un-deproteinized EPS) was 47.1% while that of the deproteinized was 45.9%. Both of un-deproteinized EPS and deproteinized EPS, by contrast, were lower than VC solution in scavenging hydroxyl radicals. When VC solution was at 0.8 mg/mL, the scavenging rate had reached 50.1%, while when the un-deproteinized EPS was at 10 mg/mL, the scavenging rate was 47.1%.

#### 3.5.2. Scavenging Ability of Superoxide Anion Radicals

**Figure 7:**
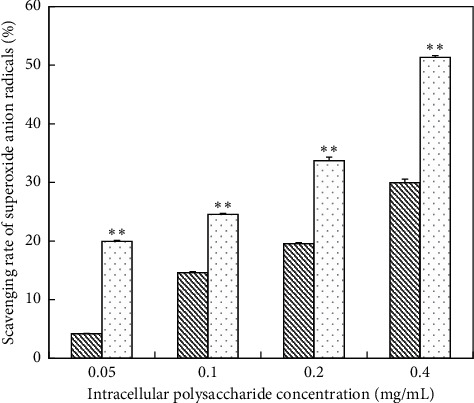
Scavenging superoxide anion radicals activity of IPS; (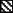) un-deproteinized IPS; (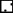) deproteinized IPS. The significance in difference between un-deproteinized IPS and deproteinized IPS was calculated by the paired-samples *T* test (^*∗∗*^*P* < 0.01).

*(1) Scavenging Ability of Superoxide Anion Radicals on IPS*. The scavenging ability of un-deproteinized IPS on superoxide anion free radicals was significantly weaker than that of the deproteinized (Figure 7). When the concentration of the IPS was 0.4 mg/mL, the scavenging rate of superoxide anion radicals (un-deproteinized IPS) was 29.98%, while the scavenging rate of deproteinized IPS was 51. 34%. As a whole, the scavenging rate of VC solution was higher than un-deproteinized IPS. The scavenging rate of VC solution was 40.5% (Table 5) when VC concentration was at 0.4 mg/mL. While the scavenging rate of deproteinized IPS was 29.98% at the same concentration. With regard to deproteinized IPS, the scavenging rate (51.34%) was slightly higher than VC.

**Figure 8:**
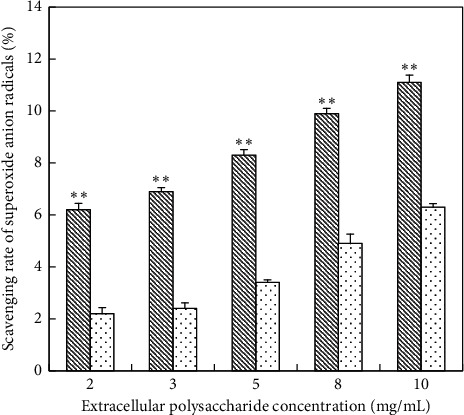
Scavenging superoxide anion activity of EPS; (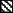) un-deproteinized EPS; (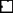) deproteinized EPS. The significance in difference between un-deproteinized EPS and deproteinized EPS was calculated by the paired-samples *T* test (^*∗∗*^*P* < 0.01).

*(2) Scavenging Ability of Superoxide Anion Radicals on EPS*. On the contrary, un-deproteinized EPS showed better ability to scavenge superoxide anion radicals than deproteinized EPS (Figure 8). The scavenging rate increased significantly when the concentration of EPS was in the range of 0–2 mg/mL and slowly at a concentration range from 2 to 10 mg/mL. At a concentration of 2 mg/mL, the scavenging rate was 6.2% (the un-deproteinized), 2.2% (the deproteinized) while the scavenging rate reached 11.1% (un-deproteinized EPS) and 6.3% (deproteinized EPS) at a concentration of 10 mg/mL, respectively. Moreover, the scavenging ability of EPS on superoxide anion radicals was obviously lower than that of the positive controls (Table 5).

#### 3.5.3. Reducing Power Determination

**Figure 9:**
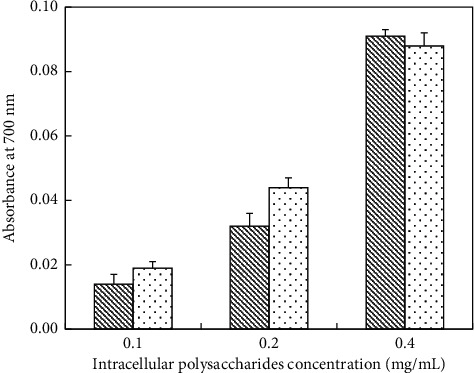
Reducing power of IPS; (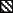) un-deproteinized IPS; (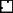) deproteinized IPS. The significance in difference between un-deproteinized IPS and deproteinized IPS was calculated by the paired-samples *T* test (^*∗∗*^*P* < 0.01).

*(1) Determination of Reducing Power of IPS*. Reducing power value of IPS appeared in two manners with increased concentrations (Figure 9). In a certain range of polysaccharides concentration, the reducing power of deproteinized IPS was stronger than un-deproteinized IPS. At a concentration of 0.2 mg/mL, the reducing power of deproteinized IPS was 0.044 while that of un-deproteinized IPS was 0.032. Instead, the reducing power of deproteinized IPS was 0.088 at a concentration of 0.4 mg/mL, while that of un-deproteinized IPS was a little higher (0.09). However, at a concentration of 0.05 mg/mL, the antioxidant ability was too small to be detected because of the lower yield. In the mass, the reducing power of VC was clearly higher than that of un-deproteinized IPS and deproteinized IPS (Table 6).

**Figure 10:**
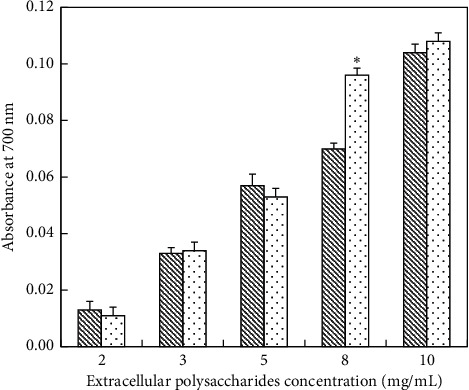
Reducing power of EPS; (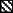) un-deproteinized EPS; (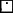) deproteinized EPS. The significance in difference between un-deproteinized EPS and deproteinized EPS was calculated by the paired-samples *T* test (^*∗*^*P* < 0.05).

*(2) Determination of Reducing Power of EPS*. As the concentration of the EPS was increased, the reduction power of the deproteinized and un-deproteinized EPS was gradually enhanced (Figure 10). At a concentration range from 0 mg/mL to 5 mg/mL, there was little difference in the reducing power between two kinds of EPS. At a concentration of 8 mg/mL, the reducing power of the deproteinized (0.096) and un-deproteinized (0.07) EPS was significantly different. At a concentration range from 6 mg/mL to 10 mg/mL, the reducing power of un-deproteinized EPS was weaker than that of the deproteinized protein. At 10 mg/mL, the reducing powers of deproteinized and un-deproteinized EPS were 0.104 and 0.108, respectively. Compared with VC positive control, the reducing powers of un-deproteinized and deproteinized EPS were wesker (Table 6).

#### 3.5.4. DPPH Radical-Scavenging Activity

**Figure 11:**
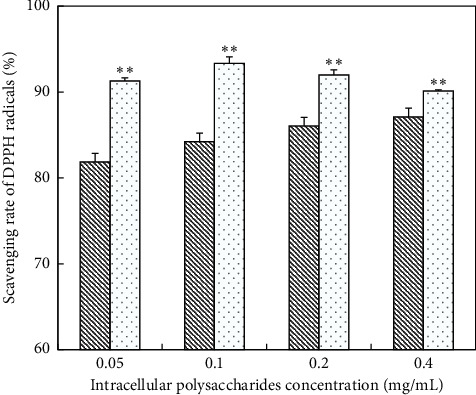
Scavenging DPPH free radicals activity of IPS; (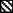) un-deproteinized IPS; (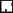) deproteinized IPS. The significance in difference between un-deproteinized IPS and deproteinized IPS was calculated by the paired-samples *T* test (^*∗∗*^*P* < 0.01).

*(1) Scavenging Ability of DPPH Free Radicals on IPS*. The ability of deproteinized IPS on scavenging DPPH free radicals was stronger than that of un-deproteinized EPS (Figure 11). With regard to the un-deproteinized IPS, the scavenging rate was increased from 81.87% to 87.12%, dramatically from 0.05 mg/mL to 0.4 mg/mL. As for the deproteinized IPS, the scavenging rate reached 93.35% at a concentration of 0.1 mg/mL. However, lower concentration of VC demonstrated highest scavenging ability (Table 7).

**Figure 12:**
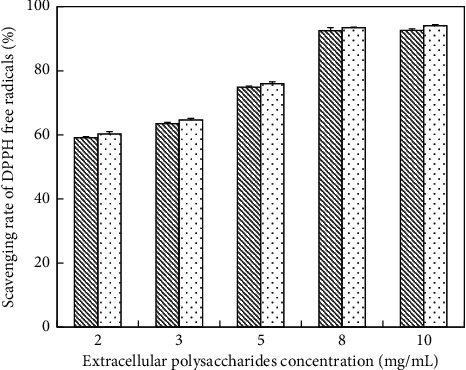
Scavenging DPPH free radical activity of EPS; (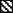) un-deproteinized EPS; (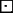) deproteinized EPS. The significance in difference between un-deproteinized EPS and deproteinized EPS was calculated by the paired-samples *T* test (^*∗*^*P* < 0.05).

*(2) Scavenging Ability of DPPH Free Radicals on EPS*. Obviously, there was no significant difference between un-deproteinized EPS and deproteinized EPS (Figure 12). The scavenging rate of the un-deproteinized reached 92.6% while that of the deproteinized was 94.1% at a concentration of 10 mg/mL. In general, the results showed that the scavenging ability of polysaccharides against DPPH free radicals was not as good as that of VC positive control (Table 7).

## 4. Discussion

Submerged cultivation is a promising and still underexplored alternative for the extraction of bioactive molecules in short time [22, 23], and large scale production under carefully monitored and controlled conditions is required for economic and scientific reasons [24]. According to the yield of polysaccharides, the optimized aeration rate for liquid fermentation was 4.5 L/min. The results showed that optimal aeration rate was helpful for polysaccharides yield of *Hygrophoropsis* sp., resulting in sufficient oxygen supply, which is frequently found in other fungal fermentation [25–27].

The results of antioxidation showed that the ability of reducing power, scavenging superoxide anion, and scavenging DPPH free radicals of deproteinized IPS was stronger than that of un-deproteinized IPS. However, the ability of IPS on scavenging DPPH radicals was lower than that of VC control. The ability of scavenging hydroxyl radicals was weaker than that of the un-deproteinized. These antioxidant capabilities are all considered to mainly be linked with the polysaccharides components of the extracts, resulting in significant medicinal efficacy, thus supporting the idea of using such compounds as active ingredients in functional products.

Though peptide from extracts showed antioxidant and anticancer activities, the extracts containing impurities like proteins may greatly limit their application in the fields of medicine and food, such as serious allergic reactions [28–30]. But proteins cannot be completely removed due to the intensive binding of some proteins to polysaccharides and the existence of glucoprotein or proteoglycan [31]. Here, deproteinized extracts showed stronger antioxidant activities than those of un-deproteinized in three assays tested, which demonstrated efficiency extracting method making little loss for bioactivity or protein making no effort for antioxidant activities.

The components having antioxidation activity in *Hygrophoropsis* sp. are unknown. As demonstrated here, polysaccharide could be one of the candidates that possessed the antioxidation activity; at least, such activity was enriched along together with the enrichment of polysaccharide in water extract derived from cultured *Hygrophoropsis* sp. mycelia. However, the exact identity of polysaccharide having antioxidation activity is not known. Additionally, the antioxidation activity could be due to other constituents that were enriched together with polysaccharides. At present, we are trying to purify further the enriched polysaccharides and to evaluate the identity of the active compound. Since by removing the bulk of polysaccharides from cultured *Hygrophoropsis* sp. mycelia the antioxidant activity (data not shown) was lost, it can be suggested that the polysaccharide is the key component exhibiting the antioxidation activity at least in part. This result is the same as *Cordyceps* [1]. By enrichment of the polysaccharide from cultured *Hygrophoropsis* sp. mycelia, the grade of antioxidation activities obtained was different, which suggested that several forms of polysaccharides might have different potency in the tested assays.

Compared with the deproteinized EPS, the ability of un-deproteinized EPS on scavenging hydroxyl radicals and superoxide anion was increased, while the un-deproteinized EPS was less effective in the reducing power and scavenging DPPH free radicals. In addition, the ability of EPS to scavenge DPPH radicals (weaker than that of the VC control) is the strongest. The results showed that the protein in fermentation liquid extracts had a certain effect on the oxidation resistance of the polysaccharides. This result is the same as polysaccharide-protein complex (PSK) that has been obtained from the mycelium of *Coriolus versicolor* with a higher share of proteins, which were more effective in antioxidative activity to scavenge superoxide and hydroxyl radicals [32].

The IPS had higher antioxidation activities than EPS at the same amount in all the assays tested. Differences in molecular level and monosaccharide composition between IPS and EPS may be the reason that the antioxidant differences existed between them [33]. Many works of the literature reported moderate correlation between the reducing ability of Fe^3+^ and the total protein content, which may be a consequence of the presence of reductive amino acids of cysteine, methionine, and tyrosine in proteins of the extract [34, 35]. In present study, neither deproteinized EPS nor deproteinized IPS showed more effective chelators of ferrous ions than un-deproteinized polysaccharides. Thus, the amino acids in the extracts of *Hygrophoropsis* sp. may be the further interesting research.

In conclusion, the water extracts of cultured *Hygrophoropsis* sp. mycelia could be chosen for natural additives of functional products. This is the first study known to evaluate the antioxidant capacity of deproteinized and un-deproteinized extracts from mushroom mycelium. However, the relevance of these *in vitro* results must be supported by future *in vivo* studies and whether the protein part of the extract in allergens should be demonstrated especially.

## Figures and Tables

**Table 1:** Orthogonal factors and levels.

Independent variables	Coded symbols	Levels		
		1	2	3
Solid-liquid ratio	A	1 : 30	1 : 40	1 : 50
Extraction time (h)	B	1	2	3
Extraction temperature (°C)	C	85	90	95

**Table 2:** IPS extraction rates of the orthogonal test with different levels of the parameter.

Test number	*A*	*B* (h)	*C* (°C)	IPS extraction rate (%)
1	1 : 30	1	85	11.26
2	1 : 30	2	90	10.48
3	1 : 30	3	95	8.72
4	1 : 40	1	90	8.19
5	1 : 40	2	95	8.69
6	1 : 40	3	85	8.40
7	1 : 50	1	95	7.48
8	1 : 50	2	85	9.18
9	1 : 50	3	90	6.89
*K*_1_	10.15	8.98	9.61	
*K*_2_	8.43	9.45	8.52	
*K*_3_	7.85	8	8.30	
*R*	2.3	1.45	1.31	

*K*_*i*_ : the mean values of index for the factors at each level and subscript *i* denotes the specific level of the parameter, *i* = 1,2,3. *R* : the difference between maximum *K*_*i*_ and minimum *K*_*i*_ for the corresponding parameter, as follows: *R* = *K*_*i*_max − *K*_*i*_min, *i* = 1,2,3.

**Table 3:** Variance analysis for IPS extraction rates.

Source of variation	Quadratic sum	Degree of freedom	Mean square	*F* value	Significance level
Solid-liquid ration	8.60	2	4.30	87.08	0.01
Extraction time	3.26	2	1.63	33.07	0.03
Extraction temperature	2.97	2	1.48	30.04	0.03
Deviation	0.10	2	0.05		
Total	14.92				

**Table 4:** Scavenging hydroxyl radical activity of VC.

Scavenging rate of hydroxyl radical scavenging (%)	VC concentration (mg/mL)				
	0.1	0.2	0.4	0.6	0.8
VC	33.3 ± 0.88	44.7 ± 0.65	47 ± 0.78	49 ± 0.08	50.1 ± 016

Each value is a mean of three biological replicates. Data shown are mean ± standard deviation.

**Table 5:** Scavenging superoxide anion radical activity of VC.

Scavenging rate of superoxide anion radicals (%)	VC concentration (mg/mL)				
	0.1	0.2	0.4	0.6	0.8
VC	25.1 ± 1.5	31.4 ± 0.79	40.5 ± 0.83	49.3 ± 0.94	55.6 ± 0.67

Each value is a mean of three biological replicates. Data shown are mean ± standard deviation.

**Table 6:** Reducing power of VC.

Absorbance at 700 nm	VC concentration (mg/mL)				
	0.1	0.2	0.4	0.6	0.8
VC	0.23 ± 0.01	0.27 ± 0.02	0.3 ± 0.15	0.33 ± 0.01	0.38 ± 0.02

Each value is a mean of three biological replicates. Data shown are mean ± standard deviation.

**Table 7:** Scavenging DPPH free radicals activity of VC.

VC concentration (mg/mL)	0.05	0.1	0.2	0.4
Scavenging rate of DPPH radicals (%)	94.7 ± 0.87	95.4 ± 0.72	95.7 ± 0.85	96 ± 0.67

## Data Availability

The data used to support the findings of this study are available from the corresponding author upon request.
